# (3a*R*,6*S*,7a*R*)-7a-Chloro-6-methyl-2-(4-nitro­phenyl­sulfon­yl)-1,2,3,6,7,7a-hexa­hydro-3a,6-ep­oxy­iso­indole

**DOI:** 10.1107/S1600536813026329

**Published:** 2013-10-09

**Authors:** Aydın Demircan, Ersin Temel, Muhammet Kasım Kandemir, Medine Çolak, Orhan Büyükgüngör

**Affiliations:** a Department of Chemistry, Faculty of Arts and Sciences, Niĝde University, TR-51240 Niĝde, Turkey; b Ondokuz Mayıs University, Arts and Sciences Faculty, Department of Physics, TR-55139 Samsun, Turkey

## Abstract

In the title compound, C_15_H_15_ClN_2_O_5_S, the tetra­hydro­furan ring adopts an envelope conformation with the O atom as the flap. The pyrrolidine ring adopts an envelope conformation with the chlorine-substituted C atom as the flap. In the crystal, two types of C—H⋯O hydrogen bonds generate *R* ^2^_2_(20) and *R* ^4^_4_(26) rings, with adjacent rings running parallel to *ac* plane. Further C—H⋯O hydrogen bonds form a *C*(6) chain, linking the mol­ecules in the *b-*axis direction.

## Related literature

For chemical background to protecting groups, see: Greene & Wuts (1999 ▶); Romanski *et al.* (2012 ▶); Chan & White (2004 ▶); Yasushi & Higuchi (2006 ▶); Blanc & Bochet (2007 ▶); Demircan & Parsons (2002 ▶); Demirtaş *et al.* (2002 ▶); Katritzky *et al.* (2004 ▶); Merlin *et al.* (1988 ▶); Büyükgüngör *et al.* (2005 ▶); Koşar *et al.* (2006*a* ▶,*b* ▶); Karaarslan *et al.* (2007 ▶); Demircan *et al.* (2011 ▶); Temel *et al.* (2011 ▶, 2012 ▶). For puckering parameters, see: Cremer & Pople, (1975 ▶). For hydrogen-bond motifs, see: Bernstein *et al.* (1995 ▶).
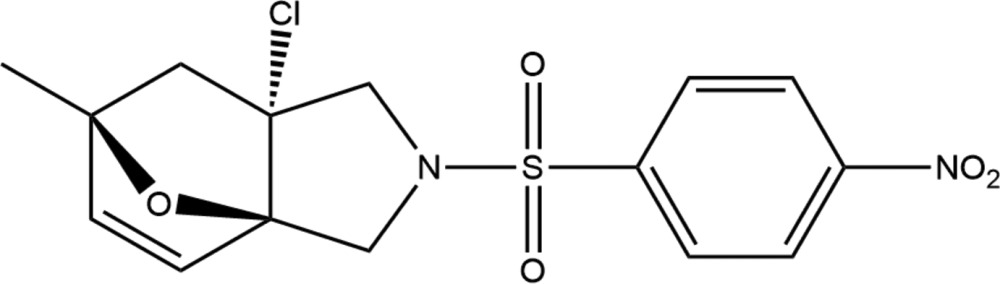


## Experimental

### 

#### Crystal data

C_15_H_15_ClN_2_O_5_S
*M* *_r_* = 370.80
Monoclinic, 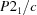

*a* = 8.6049 (5) Å
*b* = 7.1949 (3) Å
*c* = 26.8195 (15) Å
β = 101.186 (4)°
*V* = 1628.89 (15) Å^3^
*Z* = 4
Mo *K*α radiation
μ = 0.39 mm^−1^
*T* = 296 K
0.48 × 0.24 × 0.02 mm

#### Data collection

Stoe IPDS 2 diffractometer
Absorption correction: integration (*X-RED32*; Stoe & Cie, 2002 ▶) *T*_min_ = 0.894, *T*_max_ = 0.992
13941 measured reflections
3449 independent reflections
1564 reflections with *I* > 2σ(*I*)
*R*_int_ = 0.090

#### Refinement

*R*[*F* ^2^ > 2σ(*F* ^2^)] = 0.068
*wR*(*F* ^2^) = 0.192
*S* = 0.89
3449 reflections
217 parameters
H-atom parameters constrained
Δρ_max_ = 1.12 e Å^−3^
Δρ_min_ = −0.37 e Å^−3^

### 

Data collection: *X-AREA* (Stoe & Cie, 2002 ▶); cell refinement: *X-AREA*; data reduction: *X-RED32* (Stoe & Cie, 2002 ▶); program(s) used to solve structure: *SHELXS97* (Sheldrick, 2008 ▶); program(s) used to refine structure: *SHELXL97* (Sheldrick, 2008 ▶ ); molecular graphics: *ORTEP-3 for Windows* (Farrugia, 2012 ▶) and *Mercury* (Macrae *et al.*, 2006 ▶); software used to prepare material for publication: *WinGX* (Farrugia, 2012 ▶).

## Supplementary Material

Crystal structure: contains datablock(s) I, global. DOI: 10.1107/S1600536813026329/bt6935sup1.cif

Structure factors: contains datablock(s) I. DOI: 10.1107/S1600536813026329/bt6935Isup2.hkl

Supplementary material file. DOI: 10.1107/S1600536813026329/bt6935Isup3.cml

Additional supplementary materials:  crystallographic information; 3D view; checkCIF report

## Figures and Tables

**Table 1:** Hydrogen-bond geometry (Å, °)

*D*—H⋯*A*	*D*—H	H⋯*A*	*D*⋯*A*	*D*—H⋯*A*
C3—H3⋯O3^i^	0.93	2.46	3.205 (6)	138
C7—H7*A*⋯O2^ii^	0.97	2.51	3.219 (6)	129
C9—H9⋯O4^iii^	0.93	2.52	3.388 (6)	155

Symmetry codes: (i) 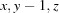; (ii) 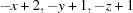; (iii) 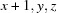.

**Fig. 1.**
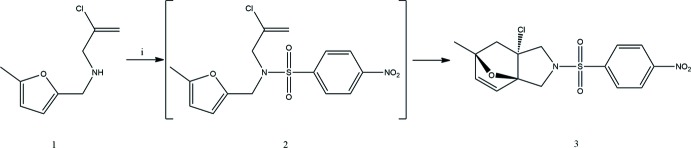
Synthesis of the title compound.

**Fig. 2.**
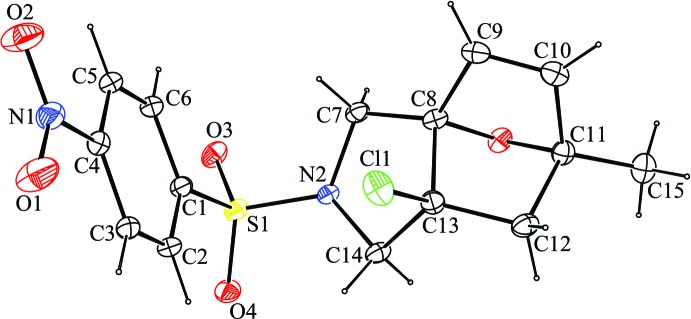
The molecular structure of the title compound, with displacement ellipsoids drawn at the 30% probability level.

**Fig. 3.**
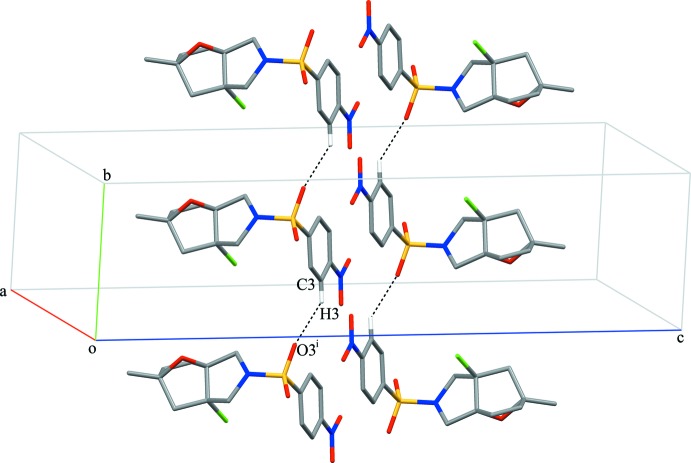
Part of the crystal structure of the title compound, showing the formation of C(6) chain.

**Fig. 4.**
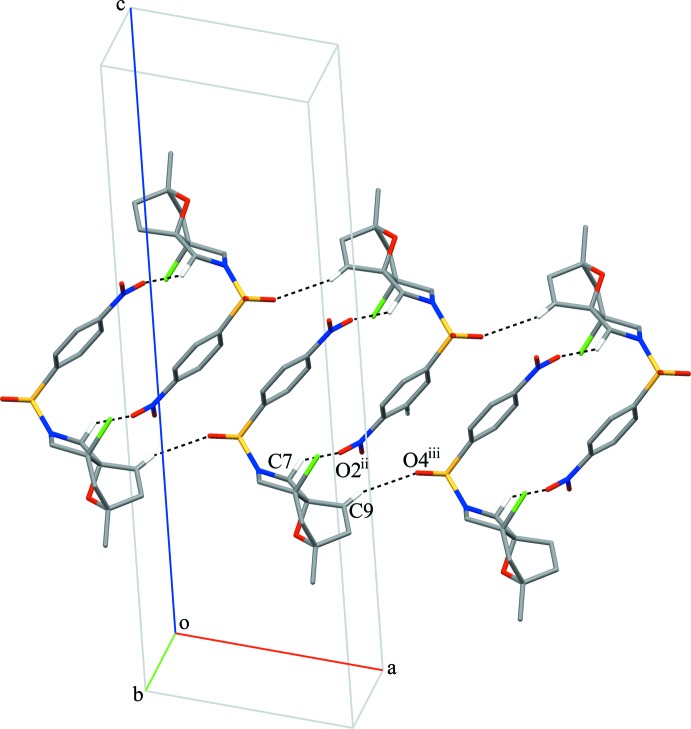
Part of the crystal structure of the title compound, showing the formation of R22(10) and R44(26) rings.

## supplementary crystallographic information

### 1. Comment

The use of protective groups has been popular in synthetic pathways (Romanski *et al.*, 2012; Chan & White, 2004; Yasushi and Higuchi, 2006; Blanc and Bochet, 2007). Many protective groups have been, developed to block other reactive sites of a molecule like NH, OH, SH, aldehyde *etc*. They should be easily removable (Greene & Wuts, 1999). Bulky protective groups on nitrogen like *tert*-butoxy carboxylate (Demircan and Parsons, 2002), the trityl group (Demirtaş *et al.*, 2002), tosyl (Katritzky *et al.*, 2004), mesyl (Merlin *et al.*, 1988) predominates in cycloaddition reactions over relatively small protective groups such as methyl, ethyl groups.

We have been working on intramolecular Diels Alder reaction (IMDAF) of compounds with a furan core using different side chains containing a heteroatom like oxygen, sulfur and nitrogen. Isoindole derivatives have been often synthesized and analyzed in our group using bulky protective groups (Büyükgüngör *et al.*, 2005; Koşar *et al.*, 2006*a*; Koşar *et al.*, 2006*b*; Karaarslan *et al.*, 2007; Demircan *et al.*, 2011). Tosyl and mesyl groups have been previously used and reported in a series of sulfonamides (Temel *et al.*, 2012; Temel *et al.*, 2011). We now here report our further finding that cycloadduct, 3 with *p*-nosyl group was generated at the intermediate *via*, 2 in aqueous condition without any other solvent system.

The title compound contains epoxyisoindole and phenyl rings linked through N—S—C bridge (Fig. 2). Tetrahydrofuran rings, pyrrolidine ring and six-membered ring that generate epoxyisoindole moiety are puckered. Both tetrahydrofuran rings adopt an envelope conformation with the puckering parameters of Q=0.516 (5) Å, φ=182.8 (6)° for O_5_/C_8–11_ and Q=0.611 (5) Å, φ=3.5 (5)° for O_5_/C_8,13,12,11_, respectively. The other five-membered ring, pyrrolidine, has the puckering parameters of Q=0.288 (5) Å and φ=277.2 (9)°. The six-membered ring, C_8–13_, has a boat conformation, according to the puckering parameters [Q=0.928 (5) Å, θ=87.5 (3)° and φ=180.8 (3)°] (Cremer & Pople, 1975).

The crystal packing of is stabilized by C—H···O hydrogen bonds. While the C7—H7A···O2 hydrogen bonds generate *R*^2^_2_(20) rings, the combination of C7—H7A···O2 and C9—H9···O4 hydrogen bonds generate *R*^4^_4_(26) rings (Fig. 4). These adjacent rings are running parallel to *ac*-plane. Additionally, C3—H3···O3 hydrogen bonds forming a C(6) chain link the molecules through *b* axis (Fig 3) (Bernstein *et al.* 1995).

### 2. Experimental

2-Chloro-*N*-(furan-2-ylmethyl)prop-2-en-1-amine, 1 (0.32 g, 1.72 mmol) in water (50 ml) was added *p*-nitrobenzenesulfonyl chloride (0.45 g, 2.06 mmol) portion wise followed by potassium carbonate (0.9 g, 6.45 mol). The reaction mixture was stirred for 48 h at 369 K. Then, the reaction mixture was allowed to warm up to room temperature and NaOH 10% (35 ml) was added. The mixture was then extracted with ethyl acetate (3x35 ml) and brine (35 ml). Combined organic phases were dried over magnesium sulfate, filtered and evaporated. The purification by column chromatography afforded colourless crystals (0.42 g, 66% yield). t.l.c., (Hexane:Ethylacetate); (8:2), Rf: 0.42). Recrystallization was performed in DCM:Hexane. Melting Point: 444–445 K.

### 3. Refinement

H atoms were positioned geometrically and treated using a riding model, fixing the bond lengths at 0.97, 0.98 and 0.93 Å for CH_2_, CH and CH(aromatic), respectively. The displacement parameters of the H atoms were constrained with *U*_iso_(H) = 1.2*U*_eq_ (aromatic, methylene or methine C).

### Crystal data

**Table:**

C_15_H_15_ClN_2_O_5_S	*F*(000) = 768
*M**_r_* = 370.80	*D*_x_ = 1.512 Mg m^−^^3^
Monoclinic, *P*2_1_/*c*	Mo *K*α radiation, λ = 0.71073 Å
Hall symbol: -P 2ybc	Cell parameters from 13941 reflections
*a* = 8.6049 (5) Å	θ = 1.6–27.2°
*b* = 7.1949 (3) Å	µ = 0.39 mm^−^^1^
*c* = 26.8195 (15) Å	*T* = 296 K
β = 101.186 (4)°	Plate, colorless
*V* = 1628.89 (15) Å^3^	0.48 × 0.24 × 0.02 mm
*Z* = 4	

### Data collection

**Table:**

Stoe IPDS 2 diffractometer	3449 independent reflections
Radiation source: fine-focus sealed tube	1564 reflections with *I* > 2σ(*I*)
Graphite monochromator	*R*_int_ = 0.090
rotation method scans	θ_max_ = 26.8°, θ_min_ = 1.6°
Absorption correction: integration (*X-RED32*; Stoe & Cie, 2002)	*h* = −10→10
*T*_min_ = 0.894, *T*_max_ = 0.992	*k* = −9→9
13941 measured reflections	*l* = −33→33

### Refinement

**Table:**

Refinement on *F*^2^	Primary atom site location: structure-invariant direct methods
Least-squares matrix: full	Secondary atom site location: difference Fourier map
*R*[*F*^2^ > 2σ(*F*^2^)] = 0.068	Hydrogen site location: inferred from neighbouring sites
*wR*(*F*^2^) = 0.192	H-atom parameters constrained
*S* = 0.89	*w* = 1/[σ^2^(*F*_o_^2^) + (0.1022*P*)^2^] where *P* = (*F*_o_^2^ + 2*F*_c_^2^)/3
3449 reflections	(Δ/σ)_max_ < 0.001
217 parameters	Δρ_max_ = 1.12 e Å^−^^3^
0 restraints	Δρ_min_ = −0.37 e Å^−^^3^

### Special details

**Table:**

Geometry. All s.u.'s (except the s.u. in the dihedral angle between two l.s. planes) are estimated using the full covariance matrix. The cell s.u.'s are taken into account individually in the estimation of s.u.'s in distances, angles and torsion angles; correlations between s.u.'s in cell parameters are only used when they are defined by crystal symmetry. An approximate (isotropic) treatment of cell s.u.'s is used for estimating s.u.'s involving l.s. planes.
Refinement. Refinement of *F*^2^ against ALL reflections. The weighted *R*-factor *wR* and goodness of fit *S* are based on *F*^2^, conventional *R*-factors *R* are based on *F*, with *F* set to zero for negative *F*^2^. The threshold expression of *F*^2^ > 2σ(*F*^2^) is used only for calculating *R*-factors(gt) *etc*. and is not relevant to the choice of reflections for refinement. *R*-factors based on *F*^2^ are statistically about twice as large as those based on *F*, and *R*- factors based on ALL data will be even larger.

### Fractional atomic coordinates and isotropic or equivalent isotropic displacement parameters (Å^2^)

**Table:**

	*x*	*y*	*z*	*U*_iso_*/*U*_eq_	
C1	0.5887 (5)	0.4475 (7)	0.44296 (17)	0.0556 (12)	
C2	0.5543 (5)	0.2605 (7)	0.44330 (18)	0.0618 (13)	
H2	0.4667	0.2139	0.4209	0.074*	
C3	0.6482 (6)	0.1425 (7)	0.47633 (17)	0.0616 (13)	
H3	0.6257	0.0161	0.4764	0.074*	
C4	0.7759 (5)	0.2143 (7)	0.50928 (17)	0.0579 (12)	
C5	0.8113 (5)	0.4015 (8)	0.51046 (17)	0.0630 (13)	
H5	0.8979	0.4474	0.5334	0.076*	
C6	0.7182 (5)	0.5177 (7)	0.47768 (18)	0.0608 (13)	
H6	0.7405	0.6443	0.4783	0.073*	
C7	0.7168 (6)	0.6656 (7)	0.34900 (18)	0.0594 (12)	
H7A	0.7832	0.6501	0.3824	0.071*	
H7B	0.7080	0.7971	0.3410	0.071*	
C8	0.7840 (5)	0.5617 (6)	0.30954 (17)	0.0573 (12)	
C9	0.9556 (6)	0.5353 (7)	0.3091 (2)	0.0727 (15)	
H9	1.0398	0.5502	0.3363	0.087*	
C10	0.9638 (6)	0.4871 (8)	0.2628 (2)	0.0741 (15)	
H10	1.0550	0.4557	0.2510	0.089*	
C11	0.7968 (6)	0.4919 (7)	0.23212 (18)	0.0599 (12)	
C12	0.7102 (6)	0.3187 (7)	0.2476 (2)	0.0691 (14)	
H12A	0.6075	0.3012	0.2257	0.083*	
H12B	0.7730	0.2068	0.2476	0.083*	
C13	0.6946 (6)	0.3746 (6)	0.30125 (18)	0.0594 (12)	
C14	0.5329 (5)	0.4221 (7)	0.31263 (18)	0.0613 (12)	
H14A	0.4592	0.4537	0.2816	0.074*	
H14B	0.4906	0.3178	0.3286	0.074*	
C15	0.7767 (7)	0.5299 (9)	0.1765 (2)	0.0832 (17)	
H15A	0.8270	0.6456	0.1714	0.100*	
H15B	0.6659	0.5372	0.1618	0.100*	
H15C	0.8244	0.4314	0.1605	0.100*	
N1	0.8795 (6)	0.0885 (8)	0.54414 (17)	0.0793 (13)	
N2	0.5591 (4)	0.5831 (5)	0.34738 (13)	0.0522 (9)	
O1	0.8474 (6)	−0.0760 (7)	0.54284 (18)	0.1241 (18)	
O2	0.9903 (6)	0.1529 (6)	0.57330 (19)	0.1193 (17)	
O3	0.4999 (4)	0.7806 (5)	0.41540 (13)	0.0780 (10)	
O4	0.3234 (4)	0.5168 (6)	0.38233 (13)	0.0780 (11)	
O5	0.7281 (4)	0.6367 (4)	0.25862 (11)	0.0637 (9)	
S1	0.47826 (14)	0.59405 (19)	0.39663 (5)	0.0621 (4)	
Cl1	0.79058 (18)	0.2099 (2)	0.34891 (6)	0.0899 (5)	

### Atomic displacement parameters (Å^2^)

**Table:**

	*U*^11^	*U*^22^	*U*^33^	*U*^12^	*U*^13^	*U*^23^
C1	0.050 (2)	0.063 (3)	0.052 (3)	−0.006 (2)	0.007 (2)	−0.009 (2)
C2	0.055 (3)	0.072 (4)	0.054 (3)	−0.019 (2)	0.002 (2)	−0.012 (2)
C3	0.072 (3)	0.059 (3)	0.053 (3)	−0.020 (2)	0.011 (2)	−0.006 (2)
C4	0.058 (3)	0.066 (3)	0.048 (3)	−0.005 (2)	0.007 (2)	0.003 (2)
C5	0.057 (3)	0.079 (4)	0.049 (3)	−0.017 (3)	0.001 (2)	−0.004 (3)
C6	0.062 (3)	0.064 (3)	0.055 (3)	−0.015 (2)	0.009 (2)	−0.009 (2)
C7	0.067 (3)	0.049 (3)	0.058 (3)	−0.011 (2)	0.001 (2)	−0.006 (2)
C8	0.054 (3)	0.052 (3)	0.061 (3)	−0.002 (2)	−0.002 (2)	−0.004 (2)
C9	0.057 (3)	0.073 (4)	0.086 (4)	−0.014 (3)	0.007 (3)	0.000 (3)
C10	0.065 (3)	0.069 (4)	0.090 (4)	−0.007 (3)	0.018 (3)	0.001 (3)
C11	0.069 (3)	0.050 (3)	0.061 (3)	0.005 (2)	0.014 (2)	−0.003 (2)
C12	0.066 (3)	0.060 (3)	0.079 (4)	−0.002 (3)	0.007 (3)	−0.018 (3)
C13	0.070 (3)	0.045 (3)	0.066 (3)	−0.004 (2)	0.018 (2)	−0.004 (2)
C14	0.058 (3)	0.056 (3)	0.066 (3)	−0.002 (2)	0.000 (2)	−0.018 (2)
C15	0.100 (4)	0.081 (4)	0.071 (4)	0.009 (3)	0.022 (3)	0.002 (3)
N1	0.085 (3)	0.083 (4)	0.064 (3)	−0.015 (3)	−0.001 (2)	0.008 (3)
N2	0.0494 (19)	0.054 (2)	0.050 (2)	−0.0019 (18)	0.0006 (16)	−0.0083 (18)
O1	0.158 (4)	0.077 (3)	0.110 (4)	−0.019 (3)	−0.042 (3)	0.016 (3)
O2	0.111 (3)	0.097 (3)	0.122 (4)	−0.026 (3)	−0.047 (3)	0.024 (3)
O3	0.084 (2)	0.073 (2)	0.073 (2)	0.0175 (19)	0.0053 (18)	−0.0221 (19)
O4	0.0455 (18)	0.107 (3)	0.078 (2)	0.0014 (18)	0.0031 (16)	−0.012 (2)
O5	0.076 (2)	0.054 (2)	0.0566 (19)	0.0075 (16)	0.0016 (16)	0.0036 (15)
S1	0.0529 (7)	0.0696 (9)	0.0603 (7)	0.0052 (6)	0.0027 (5)	−0.0132 (7)
Cl1	0.1013 (11)	0.0687 (9)	0.1046 (12)	0.0213 (8)	0.0322 (9)	0.0296 (8)

### Geometric parameters (Å, º)

**Table:**

C1—C2	1.378 (7)	C10—C11	1.512 (7)
C1—C6	1.401 (6)	C10—H10	0.9300
C1—S1	1.761 (5)	C11—O5	1.450 (5)
C2—C3	1.372 (7)	C11—C15	1.494 (7)
C2—H2	0.9300	C11—C12	1.549 (7)
C3—C4	1.371 (6)	C12—C13	1.524 (7)
C3—H3	0.9300	C12—H12A	0.9700
C4—C5	1.380 (7)	C12—H12B	0.9700
C4—N1	1.471 (7)	C13—C14	1.520 (6)
C5—C6	1.357 (7)	C13—Cl1	1.819 (5)
C5—H5	0.9300	C14—N2	1.476 (5)
C6—H6	0.9300	C14—H14A	0.9700
C7—N2	1.474 (6)	C14—H14B	0.9700
C7—C8	1.500 (7)	C15—H15A	0.9600
C7—H7A	0.9700	C15—H15B	0.9600
C7—H7B	0.9700	C15—H15C	0.9600
C8—O5	1.460 (5)	N1—O2	1.202 (6)
C8—C9	1.492 (7)	N1—O1	1.214 (6)
C8—C13	1.545 (6)	N2—S1	1.610 (4)
C9—C10	1.302 (7)	O3—S1	1.433 (4)
C9—H9	0.9300	O4—S1	1.426 (3)
			
C2—C1—C6	119.5 (4)	C15—C11—C12	116.5 (4)
C2—C1—S1	120.1 (3)	C10—C11—C12	107.0 (4)
C6—C1—S1	120.3 (4)	C13—C12—C11	100.1 (4)
C3—C2—C1	120.5 (4)	C13—C12—H12A	111.7
C3—C2—H2	119.7	C11—C12—H12A	111.7
C1—C2—H2	119.7	C13—C12—H12B	111.7
C4—C3—C2	118.8 (5)	C11—C12—H12B	111.7
C4—C3—H3	120.6	H12A—C12—H12B	109.5
C2—C3—H3	120.6	C14—C13—C12	120.0 (4)
C3—C4—C5	121.9 (5)	C14—C13—C8	103.0 (4)
C3—C4—N1	119.3 (5)	C12—C13—C8	103.5 (4)
C5—C4—N1	118.8 (4)	C14—C13—Cl1	108.3 (3)
C6—C5—C4	119.1 (4)	C12—C13—Cl1	112.4 (3)
C6—C5—H5	120.4	C8—C13—Cl1	108.7 (3)
C4—C5—H5	120.4	N2—C14—C13	105.7 (4)
C5—C6—C1	120.2 (5)	N2—C14—H14A	110.6
C5—C6—H6	119.9	C13—C14—H14A	110.6
C1—C6—H6	119.9	N2—C14—H14B	110.6
N2—C7—C8	104.9 (3)	C13—C14—H14B	110.6
N2—C7—H7A	110.8	H14A—C14—H14B	108.7
C8—C7—H7A	110.8	C11—C15—H15A	109.5
N2—C7—H7B	110.8	C11—C15—H15B	109.5
C8—C7—H7B	110.8	H15A—C15—H15B	109.5
H7A—C7—H7B	108.8	C11—C15—H15C	109.5
O5—C8—C9	100.7 (4)	H15A—C15—H15C	109.5
O5—C8—C7	111.9 (4)	H15B—C15—H15C	109.5
C9—C8—C7	126.0 (4)	O2—N1—O1	122.8 (5)
O5—C8—C13	97.0 (3)	O2—N1—C4	118.9 (5)
C9—C8—C13	110.7 (4)	O1—N1—C4	118.4 (4)
C7—C8—C13	106.6 (4)	C7—N2—C14	111.2 (3)
C10—C9—C8	106.2 (5)	C7—N2—S1	119.9 (3)
C10—C9—H9	126.9	C14—N2—S1	121.3 (3)
C8—C9—H9	126.9	C11—O5—C8	95.8 (3)
C9—C10—C11	107.0 (5)	O4—S1—O3	120.7 (2)
C9—C10—H10	126.5	O4—S1—N2	106.86 (19)
C11—C10—H10	126.5	O3—S1—N2	106.7 (2)
O5—C11—C15	112.3 (4)	O4—S1—C1	107.7 (2)
O5—C11—C10	100.6 (4)	O3—S1—C1	107.3 (2)
C15—C11—C10	117.5 (4)	N2—S1—C1	106.8 (2)
O5—C11—C12	100.6 (4)		
			
C6—C1—C2—C3	−1.8 (7)	O5—C8—C13—Cl1	159.5 (3)
S1—C1—C2—C3	174.2 (4)	C9—C8—C13—Cl1	55.2 (5)
C1—C2—C3—C4	0.6 (7)	C7—C8—C13—Cl1	−85.1 (4)
C2—C3—C4—C5	0.7 (7)	C12—C13—C14—N2	−139.8 (4)
C2—C3—C4—N1	−178.7 (4)	C8—C13—C14—N2	−25.7 (5)
C3—C4—C5—C6	−0.8 (7)	Cl1—C13—C14—N2	89.3 (4)
N1—C4—C5—C6	178.6 (4)	C3—C4—N1—O2	−179.2 (5)
C4—C5—C6—C1	−0.4 (7)	C5—C4—N1—O2	1.4 (8)
C2—C1—C6—C5	1.7 (7)	C3—C4—N1—O1	−0.6 (8)
S1—C1—C6—C5	−174.3 (4)	C5—C4—N1—O1	180.0 (5)
N2—C7—C8—O5	83.0 (4)	C8—C7—N2—C14	5.6 (5)
N2—C7—C8—C9	−154.3 (4)	C8—C7—N2—S1	155.4 (3)
N2—C7—C8—C13	−21.9 (5)	C13—C14—N2—C7	13.2 (5)
O5—C8—C9—C10	−34.8 (5)	C13—C14—N2—S1	−136.2 (3)
C7—C8—C9—C10	−162.2 (5)	C15—C11—O5—C8	−174.7 (4)
C13—C8—C9—C10	67.1 (5)	C10—C11—O5—C8	−48.9 (4)
C8—C9—C10—C11	2.9 (6)	C12—C11—O5—C8	60.7 (4)
C9—C10—C11—O5	30.0 (5)	C9—C8—O5—C11	51.2 (4)
C9—C10—C11—C15	152.2 (5)	C7—C8—O5—C11	−172.7 (4)
C9—C10—C11—C12	−74.5 (5)	C13—C8—O5—C11	−61.6 (4)
O5—C11—C12—C13	−33.9 (4)	C7—N2—S1—O4	175.4 (3)
C15—C11—C12—C13	−155.5 (4)	C14—N2—S1—O4	−37.8 (4)
C10—C11—C12—C13	70.7 (5)	C7—N2—S1—O3	45.1 (4)
C11—C12—C13—C14	110.0 (5)	C14—N2—S1—O3	−168.1 (3)
C11—C12—C13—C8	−3.9 (5)	C7—N2—S1—C1	−69.4 (4)
C11—C12—C13—Cl1	−121.0 (4)	C14—N2—S1—C1	77.3 (4)
O5—C8—C13—C14	−85.8 (4)	C2—C1—S1—O4	28.7 (5)
C9—C8—C13—C14	169.9 (4)	C6—C1—S1—O4	−155.3 (4)
C7—C8—C13—C14	29.7 (5)	C2—C1—S1—O3	160.1 (4)
O5—C8—C13—C12	39.9 (4)	C6—C1—S1—O3	−23.9 (4)
C9—C8—C13—C12	−64.4 (5)	C2—C1—S1—N2	−85.8 (4)
C7—C8—C13—C12	155.3 (4)	C6—C1—S1—N2	90.2 (4)

### Hydrogen-bond geometry (Å, º)

**Table:**

*D*—H···*A*	*D*—H	H···*A*	*D*···*A*	*D*—H···*A*
C3—H3···O3^i^	0.93	2.46	3.205 (6)	138
C7—H7*A*···O2^ii^	0.97	2.51	3.219 (6)	129
C9—H9···O4^iii^	0.93	2.52	3.388 (6)	155

Symmetry codes: (i) *x*, *y*−1, *z*; (ii) −*x*+2, −*y*+1, −*z*+1; (iii) *x*+1, *y*, *z*.
